# Lumbosacral injuries in elite Paralympic athletes with limb deficiency: a retrospective analysis of patient records

**DOI:** 10.1136/bmjsem-2020-001001

**Published:** 2021-01-15

**Authors:** Nicola R Heneghan, Esther Collacott, Paul Martin, Simon Spencer, Alison Rushton

**Affiliations:** 1 Centre for Precision Rehabilitation for Spinal Pain (CPR Spine), School of Sport, Exercise and Rehabilitation Sciences, University of Birmingham, Birmingham, UK; 2 Physiotherapy, Birmingham Royal Ballet, Birmingham, UK; 3 Physiotherapy, The English Institute of Sport, London, UK; 4 Physiotherapy, The English Institute of Sport, Newport, UK; 5 School of Physical Therapy, Western University, London, Ontario, Canada

**Keywords:** disability, lumbar spine, elite performance, sporting injuries

## Abstract

**Background:**

Compared to injury data in able-bodied athletes, relatively little literature exists for Paralympic athletes. Injury data underpins the design and evaluation of injury prevention strategies in elite sport. The aim of this study was to investigate frequency, characteristics and management of lumbosacral injuries in elite athletes with limb deficiency.

**Methods:**

A retrospective analysis of injuries in elite athletes with limb deficiency (2008 to 2017) was conducted using available data extracted from The English Institute of Sport (EIS) clinical records. Eligibility criteria: funded athletes, eligible for EIS physiotherapy support with full or partial limb deficiency. Data were analysed descriptively using frequencies.

**Results:**

A total of 107 injuries from 32 athletes were included. Participants comprised 18 men (59%), from 9 sports, with mean age for index injuries of 27 years (range 18 to 38 years) and 15 with congenital limb deficiency (47%). Average number of index injuries for congenital and traumatic limb deficient groups were 13 and 19, respectively. Where injury onset was recorded (n=79), half of injuries occurred during training (40%, n=43). Arthrogenic structures accounted for 32.7% of injuries, myogenic 26.2%, with neurogenic, discogenic and osteogenic each <5%. The number of treatments delivered in each injury episode ranged from 1 to 43, with symptom resolution taking 2 to 439 days.

**Conclusion:**

Elite athletes with limb deficiency experience lumbosacral injuries predominantly involving muscles and joints. While consistency and accuracy of data recording limits definitive conclusions, findings highlight the importance of precision in recording injury data as part of surveillance to enable implementation of effective injury prevention strategies.

**Article summary**

- Elite Paralympic athletes with limb deficiency (LD) experience lumbosacral injuries, with arthrogenic and myogenic complaints most commonly reported.
- For injury surveillance of lumbosacral injuries in elite Paralympic athletes with LD, documentation could usefully include factors such as training intensity, frequency and duration.
- Equipment changes need to be carefully managed with sufficient time for physiological and physical adaptations to take place.

## Background

Lumbosacral injuries, which can cause low back pain (LBP) are commonly experienced by elite athletes, including Paralympic athletes.^1^ According to the International Olympic Committee, the ‘lumbosacral injuries’ diagnostic code includes ‘tissue damage or other derangement of normal physical function due to participation in sports, resulting from rapid or repetitive transfer of kinetic energy’.^2^ The 1-year prevalence of LBP is 75% in elite athletes, and ranges from 24% to 66% across Olympic disciplines.^3^ Such data underpins targeted management and injury prevention strategies, although this is lacking for athletes with disabilities, despite increased participation and the emergence of the Paralympic movement.^4^ Notwithstanding the additional physical demands for those engaged in elite sport,^3^ LBP is a well-known secondary disability in individuals with lower limb amputation. Irrespective of level of amputation this is estimated as 52% to 67%^5 6^ with more than two-thirds of individuals with amputation reporting moderate-to-severe intensity^5^ and interference in function.

Specific injury data in Paralympic athletes is sparse^7 8^ with poor methodological quality and heterogeneity impacting on its utility. While data collected during the Rio 2016 Paralympic Games reported that lumbosacral injuries (lumbar spine/pelvis/buttock) accounted for 8.6% of injuries, these time-limited data collected during a competition period are not representative^8^ nor sufficient to inform targeted injury prevention strategies. Unpublished data from the English Institute of Sport’s (EIS) Illness and Injury Prevention Project between 2015 and 2018 found that the lumbosacral region was the second most injured site for Paralympic athletes.^9^ Additionally across all British athletes in the Paralympic system, those with limb deficiency (LD) account for the largest overall impairment category (25%).^8^ As well as being the largest impairment category, collectively they accounted for 30% of all recorded injuries.

**Figure 1:**
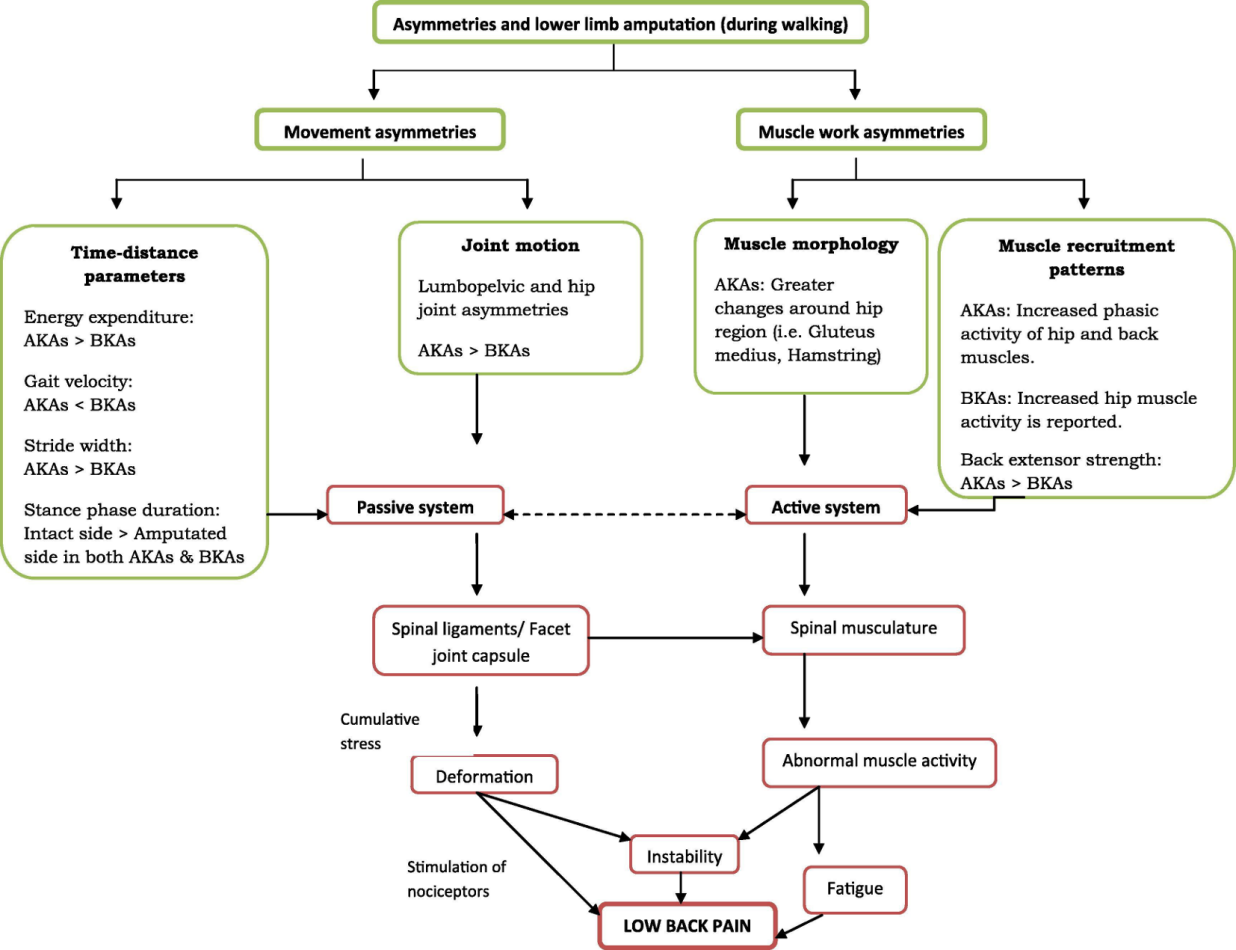
Lower limb deficiency and low back pain (Devan *et al*, 2014 with permission).^15^ AKAs, above knee amputees; BKAs, below knee amputees.

Understanding the vulnerability of this discrete athletic population is crucial in minimising their injury risk.^10 11^ Aside from the additional sports specific demands, the estimated annual prevalence of LBP in individuals with LD ranges from 50% to 90%, significantly higher than the 11% to 38% reported for general population.^12 13^ Uneven force transfer, altered joint and gait mechanics, asymmetrical movement patterns and asymmetrical limb length leading to ‘mal-adaptive’ movement impairments^12 14 15^ are some of the reported contributing biomechanical factors. These factors may also be influenced by the level of amputation and the number of limbs affected. Devan *et al*^15^ proposed a framework of contributing factors for the development of LBP in individuals with lower LD where proposed features of movement asymmetries and muscle work asymmetries are detailed. See figure 1.

Recognising the physical, physiological and biomechanical impact of LD in Paralympic athletes, injury and clinical management data (ie, techniques and approaches used, targeted tissues and so on) is required to proactively inform the development of strategies to mitigate the risk of injury. This in turn will positively influence sporting performance.^7^ For athletes with LD this may usefully consider the assessment and management of lumbosacral injuries.

### Aim

To investigate the frequency and duration to symptom resolution, characteristics and management of lumbosacral injuries in elite athletes with limb deficiency.

### Objectives

1. To identify lumbosacral injury frequency and duration to symptom resolution in elite athletes with LD, including recurrence and exacerbation.
2. To explore the aetiological factors and clinical findings of elite athletes with LD presenting with lumbosacral injuries.
3. To examine the conservative injury management of elite athletes with LD presenting with lumbosacral injuries.

## Methods

### Design

An observational research design was used, adopting a retrospective analysis of data collected from a cohort of elite athletes with LD. Data were captured from clinical records (physiotherapy and medicine) extracted from The EIS Injury and Illness Performance Project using their electronic notes systems; ‘Performance Data Management System’ (PDMS) and ‘I-Zone’.

### Inclusion criteria

All ‘elite’ athletes with LD, treated within an EIS or relevant National Governing Body setting between January 2008 and February 2017. In line with usual practice in elite sport in the UK, this includes athletes who ‘self-refer’ to a physiotherapist with a lumbosacral injury. Athletes were selected if they met specific inclusion criteria:

- National Governing Body funded to either ‘Podium’ or ‘Podium Potential’ level; therefore, deemed ‘elite’ and eligible for physiotherapy support within an EIS setting, by an EIS or National Governing Body practitioner.
- Having a LD, either upper and/or lower limb.
- All levels of LD, including part of the hand or foot.

### Injury definition

All injury records extracted from the database were classified according to an existing classification system, where stage of recovery differentiated ‘exacerbation’ from ‘reoccurrence’.^16^ For the purpose of this study, 6 months was deemed an appropriate cut-off based on tissue healing:

1. Index injury: first presentation to a physiotherapist with a complaint of lumbosacral injury.
2. Injury exacerbation: an injury of the same type, at the same site as an index injury, occurring <6 months after the index injury.
3. Injury reoccurrence: an injury of the same type and site as an index injury, occurring >6 months after the index injury.

### Data collection

Data on injuries acquired ‘directly’ or ‘indirectly’ from participation in sport^2^ were extracted from ‘I-Zone’, (patient notes repository) in the form of physiotherapy notes (2008 to 2017) including athlete injuries in the years immediately preceding the inception of ‘I-Zone’, and PDMS (2015 to 2017); a system designed by EIS to improve quality, quantity and consistency of athlete patient data management. Data were extracted from clinical notes relating to: mechanism of injury, classification according to injury site and structure (eg, joint) and clinical findings and management, including number of treatments per injury and time for resolution.

Data were anonymised and detail of individual sports removed by an independent administrator before data extraction. Data, including recorded instances of injuries sustained that predated 2008, were extracted based primarily on nature of injury, with impairment and LD (congenital or traumatic) documented to enable analysis of discrete groups. Data extraction involved three researchers (EC, PM and NH) with a staged approached to ensure accuracy; this involved initial screening of notes to inform a coding framework which was used for the subsequent analysis. Experienced researchers were involved at the each stage of data extraction.

Participants were assigned a unique identifier code to assure their identity was protected and anonymity assured.

### Data analysis

Given the use of patient notes, nature and size of the sample descriptive analysis was performed on extracted data. This included analysis of demographics, disability characteristics, injury characteristics, clinical findings, conservative management and outcomes, using mean, range, frequencies and percentages as appropriate. Histograms were used to visually display results. All data analysis was performed using SPSS V.23.

### Patient and public involvement and engagement

The study was conceived from many years of working with elite athletes with LD, in both a performance and clinical context. Findings have been discussed with physiotherapists, medical doctors, coaches and athletes with LD to inform our recommendations. Our findings have also directly informed investigation of the experiences of injury in this population and specifically health-seeking behaviours. Understanding health-seeking behaviours is needed to inform practice recommendations; education and development of guidelines.

## Results

### Participant characteristics

Data are included for 32 Paralympic athletes with LD with a lumbosacral injury (table 1). The majority presented with single LD (n=20), 10 with double LD and 2 with triple LD. The sample comprised 22 athletes (69%), who presented with a lower LD, and they accounted for 77 of the 107 injuries reported (72%). For the purpose of reporting study findings, those with double and triple LD were combined to create a multiple LD group (n=12). Sports represented by these athletes included powerlifting, para-archery, wheelchair basketball, para-cycling, para-canoe, para-triathlon, para-sailing, para-shooting or para-swimming. There were 47% (n=15) participants presenting with congenital LD (CLD), for example, dysmelia, while traumatic LD (TLD) accounted for 53% (n=17) (table 1). Athletes’ age ranged from 18 to 43 years, with the mean age for index injuries being 27 (range 18 to 38) years. This was applicable for athletes with both CLD and TLD.

**Table 1:** Participant characteristics

Impairment	Total athletes, (N)	Total injuries, (N)	Females:males, (N:N)	CLD:TLD, (N)
Total unilateral BKA	5	20	2:3	1:4
Total unilateral AKA	6	32	3:3	0:6
Total bilateral BKA	2	7	0:2	1:1
Total bilateral AKA	4	11	1:3	1:3
Total unilateral TKA	1	1	0:1	0:1
AKA and TKA	1	3	0:1	0:1
BKA and TKA	1	1	0:1	1:0
AKA and UL loss (triple)	2	2	1:1	2:0
Above elbow amputee	1	4	1:0	1:0
Below elbow amputee	6	13	4:2	5:1
Bilateral UL involvement	2	7	1:1	2:0
Unilateral hand involvement	1	6	1:0	1:0
Total	**32**	**107**	**14:18**	**15:17**

AKA, above knee amputee; BKA, below knee amputee; CLD, congenital limb deficiency; TKA, through knee amputee; TLD, traumatic limb deficiency; UL, upper limb.

### Characteristics of lumbosacral injuries

Of the 107 recorded injuries, 31% (n=33) were index (first) injuries, 45% (n=48) were a recurrence and 24% (n=26) an exacerbation. Across groups, there were 13 and 19 index injuries for those with CLD and TLD, respectively, with the percentage comparable across single and multiple LD groups (30% and 33%, respectively).

For the 79 injuries which reported timing, 40% (n=43) occurred during training, 24% (n=26) indirectly and just 8% occurred during competition (n=9) and one was ‘unclear’ (1%). Across disability groups, injury occurrences were slightly higher for the CLD group during training (63%, n=22), compared with (47%, n=20%) in the TLD group, but comparable across those with single and multiple LD (41% and 40%, respectively). Indirect injury frequency was double in the TLD group (40%, n=17) compared with 26% (n=9) in the CLD group. This was also higher in those with multiple compared with single LD, 42% and 29%, respectively.

### Injury frequency

**Figure 2:**
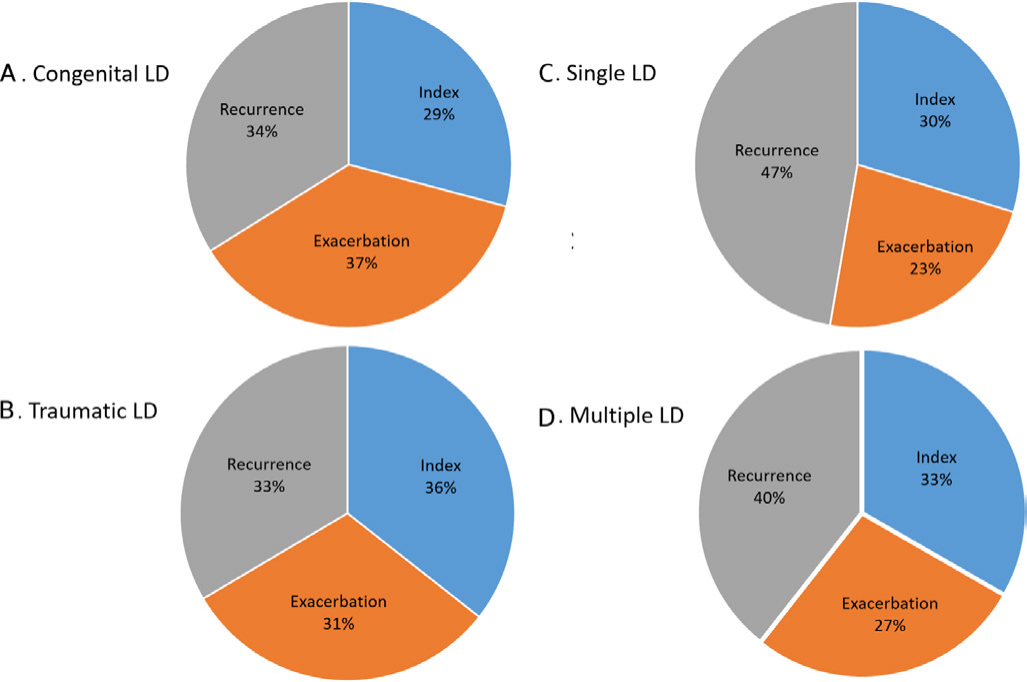
Injuries frequency across athlete groups. LD, limb deficiency.

Injuries per athlete varied considerably from one through to seven injuries, with seven athletes experiencing three injuries, and the majority (n=25) experiencing fewer than five. Seven athletes experienced more than six injuries, which was largely comparable across groups (CLD: n=3, TLD: n=4, single LD: n=4, multiple: n=3). Male and female athletes experienced a mean of 2.5 and 2.8 injuries, respectively. For CLD and TLD, frequency of exacerbations were similar although recurrences differed, with 26 (20%) injuries in the TLD group being recurrences compared with 22 (15%) in the CLD group. While single LD athletes experienced more injuries overall (69%, n=74) relative frequency for index, exacerbations were comparable. A higher number of recurrent injuries were seen in the single LD group (47%, n=35), compared with those with multiple LD (39%, n=13) (figure 2).

### Clinical diagnosis

**Figure 3:**
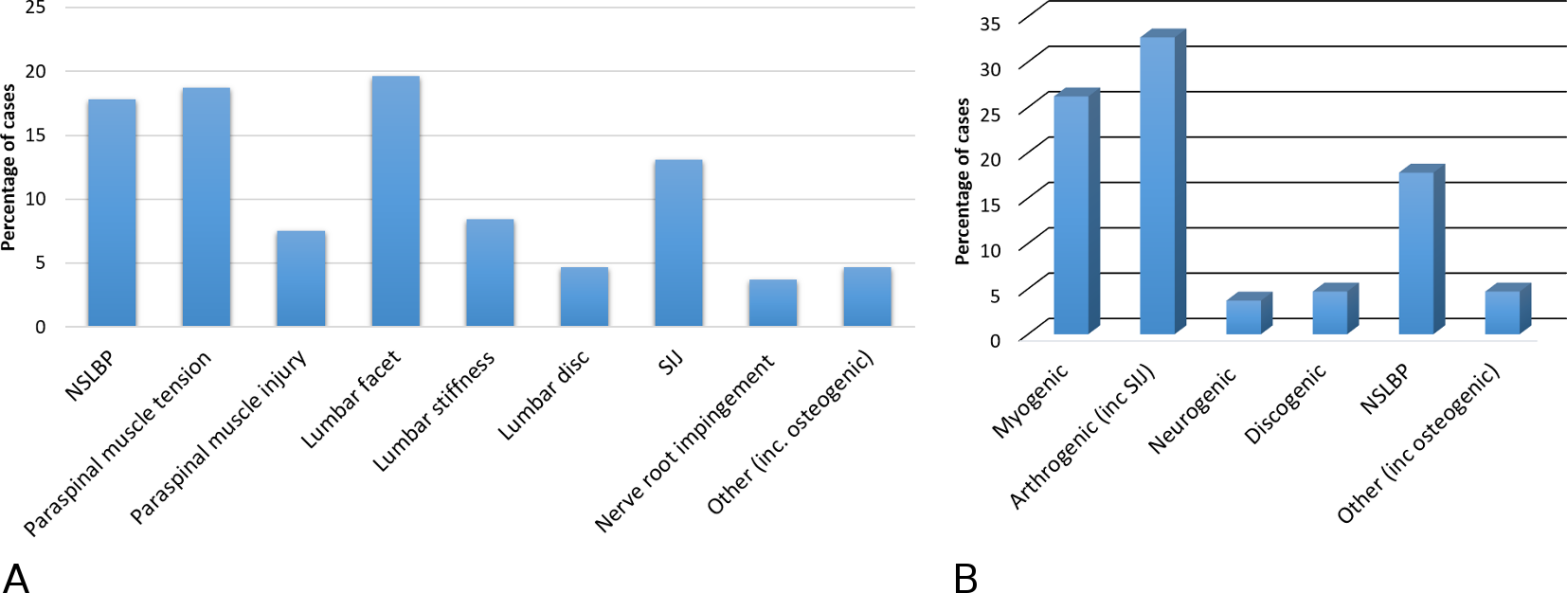
(A) Nature of clinical diagnosis. (B) Clinical diagnosis according to structure. NSLBP, non-specific low back pain; SIJ, sacroiliac joint.

Clinical diagnosis was reported according to nature of diagnosis, (figure 3A) and structure (figure 3B). Across all athletes, the most frequently documented diagnosis was lumbar facet joint (19.6%), which then contributed to arthrogenic injuries (including sacroiliac joint (SIJ)) accounting for almost one-third (32.7%) of all diagnoses. SIJ injuries accounted for 13% of all injuries. Myogenic causes, including paraspinal muscle tension or injury collectively accounted for a quarter of injuries (26.2%). Neurogenic, discogenic and other (eg, osteogenic) presentations accounted for less than 5% of injuries.

Across disability groups, multiple LD athletes experienced relatively more paraspinal muscle injuries and complaints (22%, n=16) compared with those with single LD (36%, n=12). Diagnoses of non-specific low back pain (NSLBP) however were higher in those with single LD (24%, n=17) compared with 6% (n=2) in single LD athletes. Other diagnoses were largely comparable. For CLD and TLD findings were largely comparable across all diagnoses, although there were no reported injuries involving nerve or disc in athletes with CLD.

Across injury groups almost one-third of index injuries were diagnosed as lumbar facet, with almost a half as NSLBP. A diagnosis of paraspinal muscle tension was similar for index and recurrent injuries. It is of note that no index injuries were reported for lumbar disc, nerve root impingement or other, yet exacerbations and recurrences are evident for all of these groups. The only presentation which had an increase in percentage frequency as a recurrence was SIJ (index 12% and recurrence 19%).

### Clinical findings

**Figure 4:**
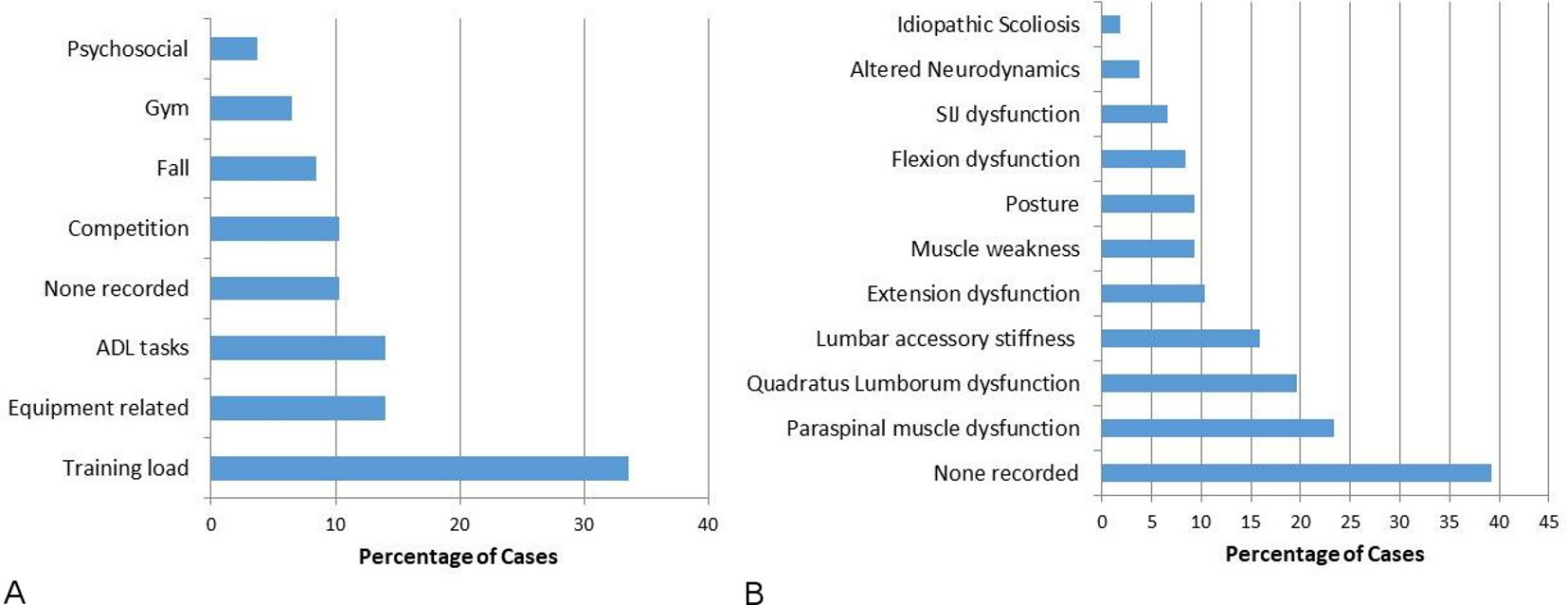
(A) Patient-reported aetiological factors. (B) Therapist-reported clinical findings. ADL, activities of daily living; SIJ, sacroiliac joint.

Patient-reported aetiological factors and therapist-reported clinical findings were explored in relation to injury onset (figure 4A) across the sample with some athletes reporting more than one factor (n=111). Notwithstanding the paucity of detail, training load was reported most frequently (34%, n=36), followed by equipment-related (22%, n=23), injuries sustained indirectly (14%, n=15), during competition (10%, n=11). Gym and falls accounted for less than 10% each and no aetiological factors were reported in 10%.

In terms of examination findings, paraspinal muscle dysfunction was reported most frequently (23%, n=25), followed by quadratus lumborum dysfunction (20%, n=21), then joint accessory movement stiffness (17%, n=16) and extension dysfunction (10%, n=11)>posture, flexion dysfunction, SIJ dysfunction, altered neurodynamics and scoliosis were reported in <10% of cases (figure 4B).

### Conservative injury management and outcome

**Figure 5:**
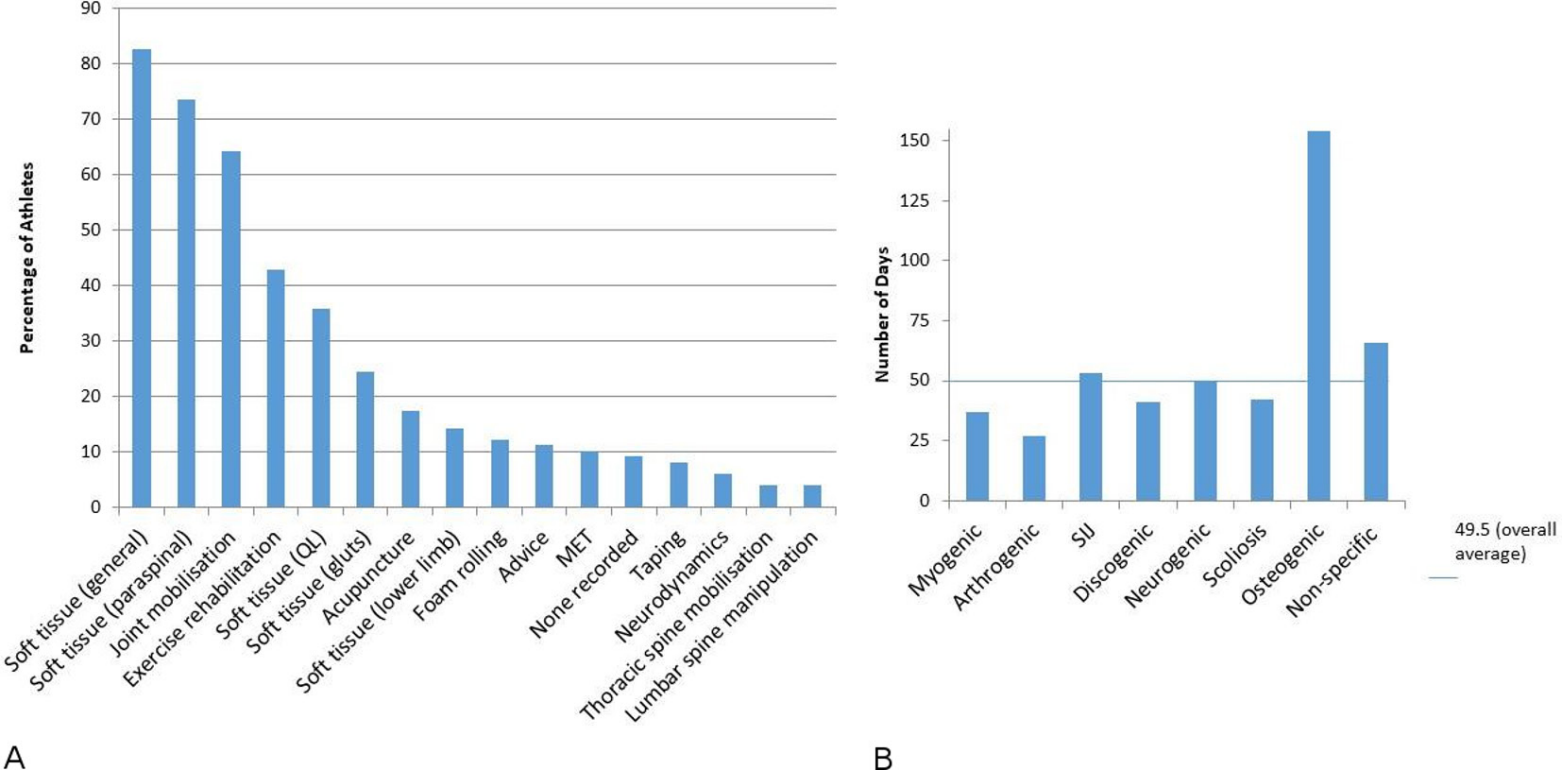
(A) Physiotherapy injury management. (B) Duration for symptom resolution according to structural diagnoses. LBP, low back pain; SIJ, sacroiliac joint.

Injury management was evaluated to examine frequency of approaches used (n=32). The majority of the athletes received soft tissue techniques, and joint mobilisation (figure 5A). Number of treatment sessions varied considerably from one (28%, n=30), two (13%, n=14), three (12%, n=13), four (9%, n=10) through to 43 sessions for one athlete. The mean number of treatment sessions was higher for the TLD group (5.8, range 1 to 13) compared with CLD 3.7 (1 to 13) sessions. There was no difference between genders, with male and female athletes receiving 4.6 (range 1 to 27) and 5.2 (range 1 to 43) sessions, respectively. Time to injury resolution was reported for 74 injuries and ranged from 2 to 439 days with a mean of 50 days; this being higher in the CLD group (66 days) and for female athletes (77 days) compared with the TLD group (39 days) and male athletes (32 days). Time for symptom resolution varied across presentation, with osteogenic presentations taking considerably longer than other presentations (figure 5b).

### Missing data

Of all 107 injury records for the 32 athletes, records had data missing. These included missing notes within physiotherapy documentation or in some instances no record of physiotherapy assessment and management. Specifically there were missing reports for timing of injury (n=28) and time to resolution (n=33).

## Discussion

This is the first report of lumbosacral injuries in elite Paralympic athletes with LD. Irrespective of gender or disability type, lumbosacral injuries constitute a significant challenge to elite Paralympic athletes with LD. Our findings are in line with other research investigating LBP in amputees^11 15^ with 68% of all Great British Paralympic athletes receiving interventions for a lumbosacral injury. Our findings provide preliminary data to support a vision for precision injury surveillance and to inform effective injury prevention strategies in this population.

### Frequency and characteristics of injury

Injury prevalence and frequency findings mirror existing literature of LBP in elite athletes^1 3^ although unlike existing literature, we detail a diagnostic typology centred on muscle and joint involvement in lumbosacral injuries. Spinal diagnostic classification has long been a challenge in clinical practice, with NSLBP being used as a ‘catch all’ for complaints in the absence of a pathoanatomical cause. Our findings reflect this with a fifth of injuries categorised as NSLBP, although a more specific clinical diagnosis was provided for some. Given the need to protect athlete identify we were unable to analyse for a potential association with sport specific spinal kinematics that have been reported as a risk factor in some able-bodied sporting disciplines (eg, rowing).^3^ Gender and age are frequently discussed confounders regarding LBP^3^ although with our relatively small sample size and the osteogenic injuries (longer healing time) occurring in female athletes, caution is needed in drawing definitive conclusions in this regard. From a previous study looking at upper quadrant injuries,^9^ a significantly higher percentage of lumbosacral injuries were either recurrent or exacerbations (69%) rather than index injuries. This finding is in line with the evidence that a previous history is a risk factor for a further episode of LBP.^3^

### Contributing factors and clinical findings

Increased training load was the most frequently reported cause of injury, which mirrors findings from able-bodied^17^ and Paralympic athletes.^9 18^ Cumulative load on spinal ligaments/facet joint capsules and spinal musculature may explain our findings, with one-third of index injuries being facet joint injuries, and muscle dysfunction the most common finding on physical examination. Our findings closely map to Devan *et al*’s framework of contributing factors^15^ to LBP in lower limb amputees. The framework makes an association between LBP and movement asymmetries (time-distance parameters and joint motion) and muscle work asymmetries (muscle morphology and muscle recruitment patterns), and mediated through cumulative loading of the passive (joint) and active subsystems (muscle) respectively.^15^ While the exact relationship between muscle dysfunction and LBP in this unique, yet heterogeneous athletic population lacks clarity, the framework details parameters which could be further incorporated into injury surveillance in the future (eg, new training programmes or equipment changes).

As well as training volume, equipment-related problems were reported. A fifth of all athletes in this study reported problems with equipment, and half of these specifically linked to equipment alterations. As well as the immediate influence this may have on movement or muscle work asymmetries, this supports a recommendation for closer monitoring of musculoskeletal adaptations when changing equipment,^19^ with graded exposure to minimise mal-adaptive movements and muscle overload.^15^

### Conservative injury management

Passive soft tissue therapies (general and specific) were most commonly used, with specific joint techniques being used less frequently. Paraspinal muscles, gluteal muscles and quadratus lumborum were specifically targeted to manage the consequences of load asymmetry and subsequent muscle tension or injury.^15^ Movement asymmetries^15^ may have arisen from both the functional demands of individual sports (eg, para-canoe vs para-swimming), and specific LD.

Although active exercise rehabilitation was reported in 39% of lumbosacral injuries, this is likely an underestimation of the use of exercise in rehabilitation, since strength and conditioning sessions were not recorded in clinical notes. Moreover the specific aims of exercise rehabilitation provided was unclear and would be needed to inform implementation of effective injury prevention strategies. Using a framework of clinical reasoning in conjunction with Devan’s model,^15^ personalised outcome focussed exercise prescription (mobility, motor control, work capacity and strength) is recommended and recognises the unique demands of individual elite sports.^20^

### Strengths and limitations

Data were drawn from the clinical notes of all elite athletes with LD during a 12-year period. Potential researcher bias was minimised with the removal of potentially identifying data, for example, sport. Findings provide preliminary evidence to support further research and clear practice recommendations.

Where data originated from different practitioners, there may have been some variability in expertise, use of diagnostic codes and accuracy of recording. Data were taken from clinical notes that often lacked sufficient detail or missing information. The main reason for missing data was treatment by a practitioner without access to either ‘PDMS’ and ‘I-Zone’; electronic patient documentation systems. Where data was derived solely from funded athletes, caution should be taken generalising findings to all elite athletes with LD. Poor reporting and lack of standardisation also precluded the assessment of injury severity, which would report ‘the number of days from date of injury to the date of return to full participation in training, and availability for match selection’.^16^ Finally blinding to individual sports precluded evaluation of injuries for specific sports.

### Practice and research recommendations

As well as endorsing earlier efforts to improve injury record keeping through the development and implementation of PDMS in 2015, our findings support other novel initiatives to benefit this population. In particular acquiring high quality epidemiological data as is underway through the ‘Sports-Related Injuries and Illnesses in Paralympic Sport Study’ (SRIIPSS) study and the recent consensus statement from the International Olympic Committee (IOC), advocating a robust methodological framework to support comprehensive recording and reporting of epidemiological data on injuries.^2^ Within lumbosacral injury surveillance data recording and research, precise information on predisposing factors to LBP is needed, such as training intensity, frequency, duration, as well as timing relative to competition, and sport specific functional movements demands, for example, excessive/repetitive spinal motion.^3^ Equipment changes need to be carefully managed with sufficient time for physiological and physical adaptations to take place. While the focus here has been on clinical findings and management, a multiplicity of other reported risk factors for LBP injury or persistence of symptoms require consideration as part of a holistic approach.^21^

## Conclusion

Elite Para athletes with LD experience lumbosacral injuries, with arthrogenic and myogenic complaints most frequently reported. Specific management approaches targeted more myogenic structures resulting from paraspinal muscle tension or injury. Findings highlight the importance of precision in injury surveillance in athletes with LD to enable implementation of effective injury prevention strategies. Knowledge should be used to inform the development of sport specific injury surveillance approaches in Paralympic sport.
